# Digital tools for training speech therapists: diagnosis of speech disorders in children

**DOI:** 10.1192/j.eurpsy.2025.1899

**Published:** 2025-08-26

**Authors:** A. Akhmetzyanova, Z. Egorova, T. Artemyeva

**Affiliations:** 1 Department of Psychology and Pedagogy of Special Education, Kazan Federal University, Kazan, Russian Federation

## Abstract

**Introduction:**

The evolution of digital technologies creates new potential in training specialists who work with children with speech development disorders (6A01.0, ICD-11). The use of digital tools, such as video cases depicting real or simulated speech disorders, provides more opportunities for students to acquire practical diagnostic skills in classroom settings before encountering real individuals with speech disorders.

**Objectives:**

Identifying the attitudes of students, future speech therapists, towards the use of classroom-based diagnostic simulation as a form of training.

**Methods:**

Sixty-two second-year students in the Speech Therapy program were surveyed. The future specialists were asked to anonymously answer open-ended questions about which training methods they consider most effective for enhancing their competencies in diagnosing children with speech disorders.

**Results:**

Forty-three students indicated that they would like to increase the number of practical hours in pre-schools and schools, where they can observe real cases of speech disorders. Thirty-nine students responded that during classroom sessions and self-study, they would like to have unlimited access to video materials showcasing the widest possible range of speech disorders.

**Conclusions:**

Future speech therapists are highly interested in the practical study of speech disorders. However, during their internships, students are often limited in their access to diverse range of examples of the disorders. Creating an educational digital resource featuring video cases which allows students to study cases of speech disorders not only during class time but also during independent work at their own convenience and pace will significantly contribute to successful development of diagnostic competencies. This paper has been supported by the Kazan Federal University Strategic Academic Leadership Program (PRIORITY-2030).

**Disclosure of Interest:**

None Declared

